# Precipitation variability increases in a warmer climate

**DOI:** 10.1038/s41598-017-17966-y

**Published:** 2017-12-21

**Authors:** Angeline G. Pendergrass, Reto Knutti, Flavio Lehner, Clara Deser, Benjamin M. Sanderson

**Affiliations:** 1 National Center for Atmospheric Research, Boulder, CO USA; 2 Institute for Atmospheric and Climate Science, ETH Zurich, CH-8092 Zurich, Switzerland

## Abstract

Understanding changes in precipitation variability is essential for a complete explanation of the hydrologic cycle’s response to warming and its impacts. While changes in mean and extreme precipitation have been studied intensively, precipitation variability has received less attention, despite its theoretical and practical importance. Here, we show that precipitation variability in most climate models increases over a majority of global land area in response to warming (66% of land has a robust increase in variability of seasonal-mean precipitation). Comparing recent decades to RCP8.5 projections for the end of the 21^st^ century, we find that in the global, multi-model mean, precipitation variability increases 3–4% K^−1^ globally, 4–5% K^−1^ over land and 2–4% K^−1^ over ocean, and is remarkably robust on a range of timescales from daily to decadal. Precipitation variability increases by at least as much as mean precipitation and less than moisture and extreme precipitation for most models, regions, and timescales. We interpret this as being related to an increase in moisture which is partially mitigated by weakening circulation. We show that changes in observed daily variability in station data are consistent with increased variability.

## Introduction

Precipitation variability is a crucial climatic factor for society, agriculture, and the environment; increased precipitation variability can reduce agricultural yield^1^, and in developing countries can affect growth of children^2^. Precipitation variability also connects extreme wet and dry events, floods and droughts, which pose threats to the environment and society^3^. Yet it is sometimes assumed that precipitation variability does not change in a warming climate [e.g., refs^4,5^], or that mean precipitation and its variability change at the same rate^6^. While temperature variability does not change systematically in response to projections of global warming in most regions^7,8^, it is not clear that this should hold for precipitation.

The reigning conventional wisdom of how precipitation changes with warming is that mean precipitation change is energetically constrained to about 2% K^−1^ ^9^, while extreme precipitation change is driven primarily by the change in near-surface moisture with little change in coincident circulation, with a magnitude of about 6% K^−1^ for the 99.9^th^ all-day percentile of daily precipitation^10^. To formulate a complete theory of how precipitation changes with warming, we must connect explanations for the mean and extremes by addressing how variability responds to warming. Remarkably few studies have undertaken this charge.

Two decades ago, early climate model simulations indicated that daily to interannual precipitation variability increases in response to a doubling of carbon dioxide^11–13^. This notion was supported by station observations showing increased variability on decadal timescales^14^ and confirmed for interannual timescales by studies with small ensembles of model simulations^15,16^. But in the mid-2000s, the changes in interannual to decadal variability were deemed slight in the face of large present-day variability^17^, and subsequent work downplayed its importance^18,19^. Very recent work has focused on changes in precipitation variability at regional scales, including the monsoons^20^, and one study has examined decadal prediction and identified increases in interannual precipitation variability^21^. However, most work in the last decade focused on the robust response of ENSO-related precipitation change across models, which occurs despite disagreement on changes in ENSO-related sea-surface temperature variability^22–27^; these studies are all limited to interannual timescales and focused on the tropics.

Here we quantify the change in precipitation variability on timescales from daily to decadal with warming in three ensembles of global climate model simulations: the Coupled Model Intercomparison Project version 5 (CMIP5) ensemble, and two large initial-condition ensembles using the CESM1 and GFDL models (see Methods for details). We use standard deviation of precipitation as a metric of its variability (following refs^4,5^; alternative metrics are discussed in the Supplementary Information). We primarily focus on land because this is where most impacts will be felt. The changes in precipitation variability are interpreted in the framework for the changing distributions of moisture, vertical velocity, and precipitation presented in ref.^28^.

## Results

### Spatially-aggregated precipitation variability change

**Figure 1:**
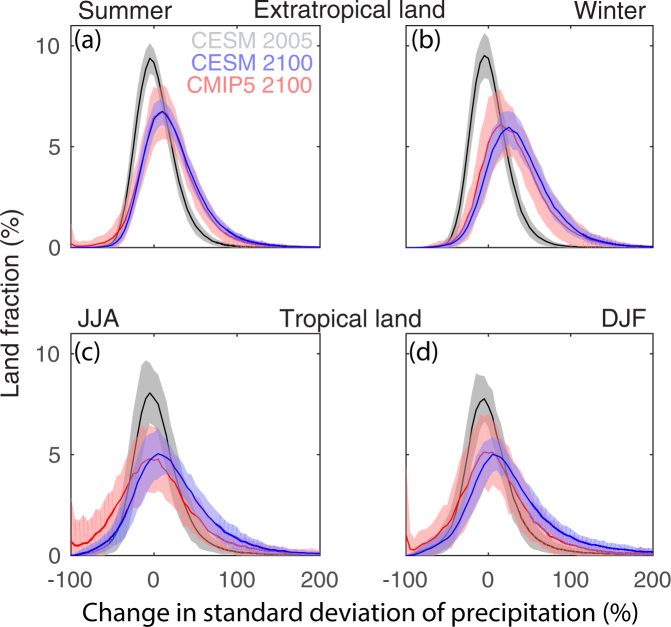
Spatially aggregated precipitation variability change. Land area fraction experiencing a given change in the interannual standard deviation of seasonal-mean precipitation over extra-tropical land in (**a**) summer and (**b**) winter, and tropical land in (**c**) JJA and (**d**) DJF at individual grid points from 1976–2005 to 2071–2100 forced by the RCP8.5 scenario, and changes expected from natural variability sampled as differences between randomly-drawn members of the CESM1 single-model ensemble for 1976–2005 (grey shading). Shading indicates the 5–95% confidence interval.

Are there important changes in precipitation variability in response to climate change? An earlier study^17^ asserted that changes in variability were not important by visual inspection of precipitation time series at individual grid points because modern precipitation variability is already large. To assess whether models predict an increase in interannual precipitation variability with warming, we examine the spatially-aggregated change^29^ in the interannual standard deviation of seasonal-mean precipitation. Spatial aggregation is useful for expressing the risk of climate impacts probabilistically over land areas, because variability at each particular grid cell is high and models do not accurately simulate the location of all precipitating features. Grid cells are binned according to their change, and the resulting histogram is plotted with the frequency represented as a fraction of land area. Figure 1 shows these histograms of the change in interannual standard deviation of precipitation by land area from each member of the CMIP5 and CESM1 ensembles from the end of the 20^th^ century to the end of the 21^st^ century. As a reference case to quantify the expected internal variability in precipitation when no change in climate occurs, the difference between members of the CESM1 ensemble drawn randomly without replacement during the historical period is also included.

Spatial aggregation of precipitation variability reveals a robust change in response to warming. The change in interannual standard deviation of precipitation is distinct from present-day internal variability. The interannual standard deviation of precipitation increases over a larger land fraction than it decreases in the CMIP5 and CESM1 ensembles in most regions, seasons, and models (see Fig. S1 for results from the GFDL ensemble; which are similar in the extratropics but have some differences in the tropics, addressed later). In the extratropics in summer, internal variability is relatively small, and variability across models is only slightly higher (see Methods for region and season definitions). Agreement across CMIP models as well as with CESM1 (and GFDL: Fig. S1) is higher over extratropical land than over tropical land. In the tropics, CESM1 shows a larger increase in variability than the CMIP5 models.

### Spatial patterns of precipitation mean, variability, and extremes

**Figure 2:**
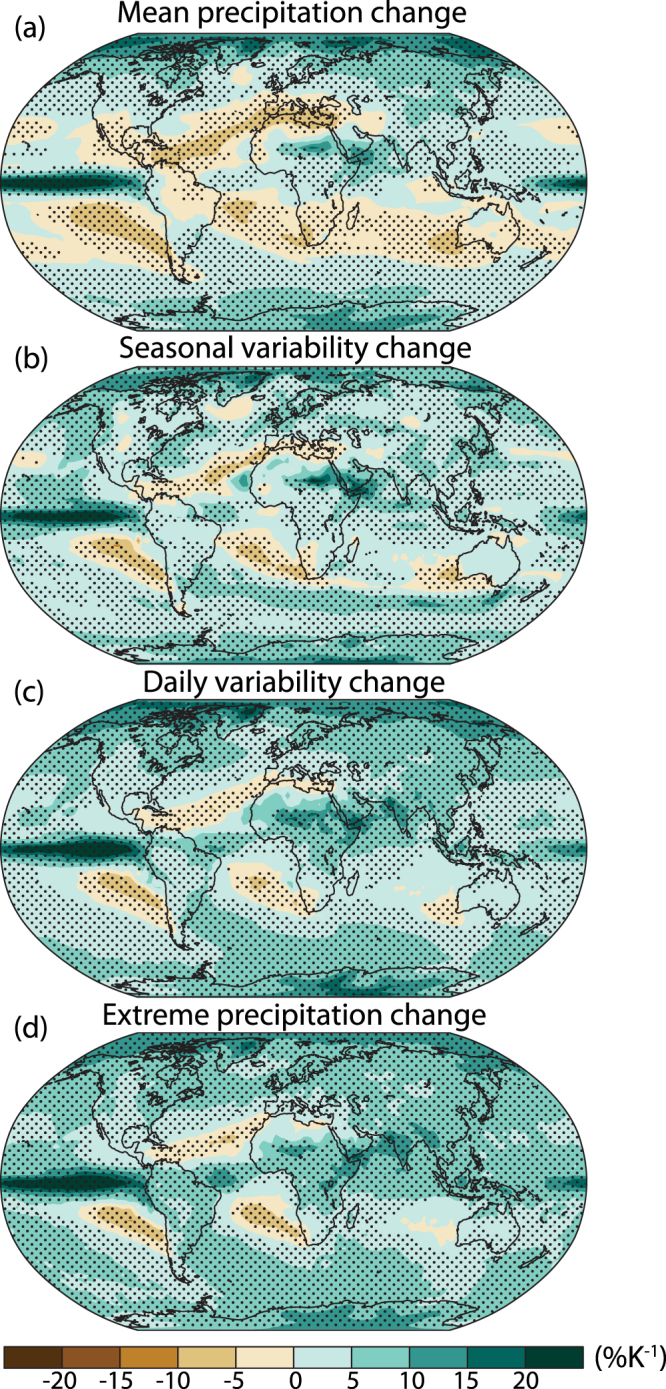
Spatial patterns of change in mean, variability and extreme precipitation. Change in precipitation (**a**) mean, (**b**) seasonal standard deviation, (**c**) daily standard deviation, and (**d**) daily maximum for the CMIP5 multi-model mean in 2071–2100 relative to 1976–2005 scaled by the change in global mean surface air temperature. Stippling indicates gridpoints where at least 67% of models agree on the sign of the change. Maps were generated using NCL^53^.

The spatial patterns of the fractional changes in mean, interannual and daily variability, and extreme precipitation share many features (Fig. 2). All show maxima in the ITCZ and monsoon regions, as well as at high latitudes of both hemispheres. This is consistent with previous research^30^ showing increasing interannual monsoon variability in response to a doubling of carbon dioxide. Mean, variability, and extreme precipitation all have minima in the subtropics, particularly the eastern ocean basins, extending to adjacent land areas. The regions of decrease are most extensive for mean precipitation (26% of land area), less for variability (7.3% of land for seasonal and 2.9% for daily) and least for extreme precipitation (1.5%).

There are also differences between the spatial patterns of changes in interannual variability and mean precipitation. The increase in variability is smaller at high latitudes than mean precipitation change, in winter in the Arctic and both winter and summer over the Southern Ocean (Fig. S2). The increase in mean precipitation in winter in the Arctic coincides with increased warming which has been attributed to sea ice loss^31^. The magnitude of changes in daily precipitation variability and mean precipitation at high latitudes are similar.

### Magnitude of precipitation variability change

By how much do climate models predict precipitation variability to change in response to changes in climate? We consider the magnitude of the change in precipitation variability with warming in the context of two null hypotheses. The first hypothesis arises from a statistical argument for a positive-definite variable like precipitation. The simplest form of change for precipitation is to scale an initial timeseries by a constant, which leads to a change in standard deviation equal to the change in its mean^11^. Our first hypothesis, then, is that the change in standard deviation follows mean precipitation. The predicted rate of increase of global-mean precipitation is energetically constrained to about 2% K^−1^ ^9^.

Instead of following the mean, extreme precipitation increases much more with warming, at a rate similar to the increase in near-surface moisture^10^. The second hypothesis is that variability is also driven by the change in near-surface moisture and would be similar in magnitude to its change^15,32^. A simple model assuming that precipitation rate is proportional to near-surface moisture and vertical velocity, which can each be represented by an appropriate distribution^28^, predicts that if moisture were to increase with no corresponding change in circulation or relative humidity, the change in standard deviation of precipitation would equal the rate of moistening, 6–7% K^−1^ (this is also noted by ref.^33^).

For the change in precipitation variability to follow the change in moisture, the circulation must not change, according to the model of ref.^28^. If precipitation rate is proportional to moisture and vertical velocity (when vertical velocity is upward), then a strengthening of the circulation (and thus the average magnitude of vertical velocity) would increase precipitation variability, while a weakening of the circulation and decrease in magnitude of vertical velocity would decrease precipitation variability. Climate models project a decrease in variability of the tropical circulation in response to warming^34^ related to an increase in stability^35–37^, and so we should expect the influence of circulation to decrease precipitation variability below the magnitude that would be predicted from the influence of moisture alone.

**Figure 3:**
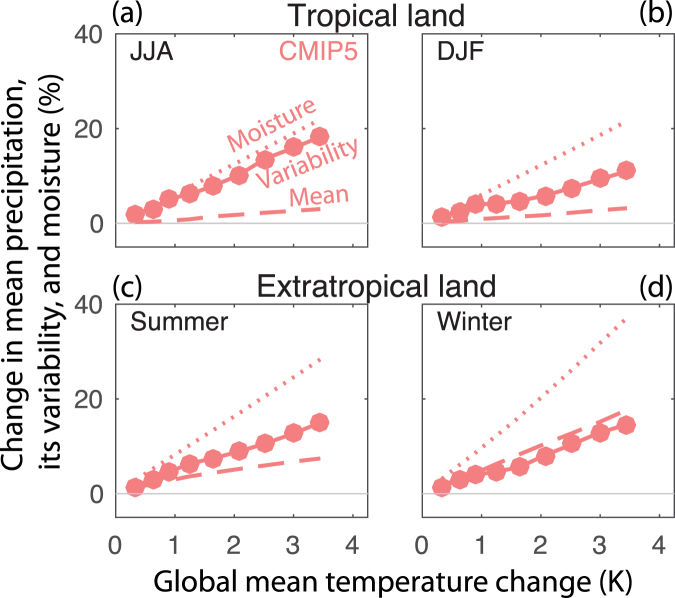
Rate of change of variability with warming. Change in seasonal mean (dashed lines), standard deviation (solid lines), and moisture (dotted lines) averaged over extra-tropical land in (**a**) summer and (**b**) winter, and tropical land in (**c**) JJA and (**d**) DJF as a function of global-mean surface temperature for the CMIP5 multi-model mean. Each marker indicates a 30-year period centered on consecutive decades between 2006 and 2086 relative to the 1976–2005 base period.

Figure 3 shows the change in spatially-averaged precipitation variability over successively warmer decades of the CMIP5 simulations (single-model ensemble results are shown in Fig. S3). The rate of change of precipitation interannual standard deviation is generally at least as large as mean precipitation and smaller than moisture for the CMIP5 multi-model mean over land (also for CESM1 and GFDL). In the tropics and extratropical summer, the increase in standard deviation is greater than the increase in mean precipitation and less than near-surface moistening. In extratropical winter it increases at a rate similar to mean precipitation change. Changes over ocean have slightly smaller magnitudes but are otherwise similar (not shown). The relationship between standard deviation and global-mean temperature is essentially linear when taken in average over large areas and ensembles of model simulations. In contrast to precipitation variability, temperature variability (Fig. S4) does not change systematically with warming, with the exception of high latitude winter where it decreases in association with sea ice decline^38,39^.

Because the magnitude of precipitation variability increase is in most cases greater than the change in mean precipitation, the first hypothesis can be rejected as an explanation. The second hypothesis, that precipitation variability is driven by moisture, can provide a starting point to explain its changes. Because in most models and regions the change in variability is smaller than the change in moisture, the simple model discussed above implies that circulation must also weaken. The mean, variability, and extreme of precipitation each measure a different aspect of the distribution of precipitation, but they are not independent of one another. In order to reconcile the differing changes in variability with extreme precipitation, there must also be a change in the shape of the distribution of circulation, because merely changing its strength does not break the symmetry between the changes in mean precipitation, variability, and the magnitude of extreme precipitation change. Reference^28^ showed that an increase in the skewness of the vertical velocity distribution reconciles these differences.

### Timescale dependence

**Figure 4:**
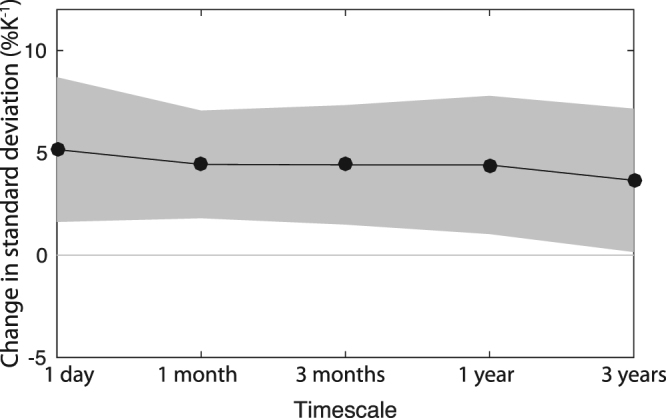
Precipitation variability change by timescale. The change in standard deviation of precipitation averaged over all land grid points divided by the change in global-mean surface air temperature in 2071–2100 relative to 1976–2005 in the CMIP5 multi-model mean for time scales ranging from 1 day to 3 years (note that the value at 3 years represents the change in 2050–2100 relative to 1955–2005; see text for details). Gray envelope denotes the 95% confidence interval according to a two-tailed student’s *t-*test.

Do the magnitude of and mechanisms driving changes in precipitation variability depend on the timescale considered? Fig. 4 shows the change in variability of precipitation at temporal resolutions from 1 day to 3 years averaged over all land grid points for CMIP5 (Fig. S5 shows CESM1 and GFDL, extending to multi-decadal time periods; Fig. S6 shows the change in power spectral density). Changes in precipitation variability averaged over global ocean grid points are similar, with somewhat smaller magnitude; we primarily focus on land because this is where most impacts on humans will be felt, but we do not expect the dominant mechanisms for change to differ substantially over ocean.

The magnitude of precipitation variability change varies remarkably little across this wide range of timescales, maintaining an increase between 4 and 5% K^−1^ in the CMIP5 multi-model global land mean, with a modest decrease in magnitude toward longer timescales. The global average (including ocean) has a similar pattern with a slightly smaller increase, 3–4% K^−1^. The smaller increase over ocean might be related to the smaller warming there^40^, to soil-moisture feedbacks over land^41^, or to compensating regional changes over ocean.

A notable feature of the precipitation variability change is that no individual timescale stands out, including ENSO timescales (>3 y; see also Fig. S6). Recent work shows that the ENSO-related precipitation increases in magnitude with warming, maintaining a similar spatial pattern^22–24,27^. The consistent response of precipitation variability across timescales indicates that this reflects processes whose influence is felt on all timescales, like the increase in moisture, rather than processes related predominantly to ENSO. Indeed, if we think of precipitation as a red-noise process, the effects of moisture on the change in the characteristics of this process project onto all timescales. That said, ENSO does modulate the *difference* in interannual precipitation variability *among* models, particularly in the tropics in DJF. The CESM1 and GFDL models happen to bracket the responses of precipitation variability across all CMIP5 models (Figs 3 and S3); but the GFDL model has an ENSO variability response that is an outlier among CMIP5 models^42^. Removing the influence of ENSO by isolating neutral ENSO years brings the change in precipitation variability in these two models into good agreement (Fig. S7).

The uncertainty in precipitation variability due to internal variability alone (Fig. S5) is small for short timescales and increases dramatically moving to longer timescales. This stands in contrast to the uncertainty across the CMIP5 multi-model ensemble, which is relatively large at all timescales. This indicates large structural uncertainty at short timescales, and that changes in short timescale variability would be detectable in the observational record before changes in variability at longer timescales.

### Observed daily precipitation variability change

**Figure 5:**
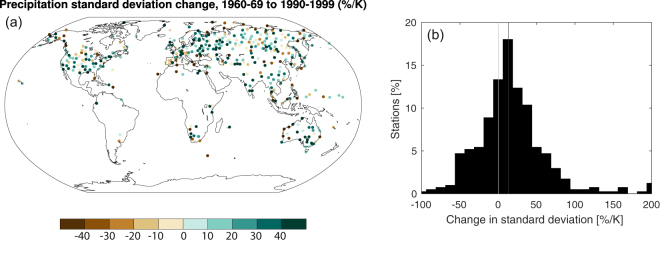
Observed daily precipitation variability change. Change in the standard deviation of daily precipitation from 1960–1969 to 1990–1999 per degree global mean surface temperature change, (**a**) at station locations and (**b**) aggregated as a histogram. Dark gray line in (**b**) is the median change across stations. See Methods for details. The map was generated using NCL^53^.

Are changes in precipitation variability already occurring? Daily precipitation variability can be estimated with greater certainty than interannual precipitation variability because the number of samples during the period of observations is larger (Fig. S5 shows uncertainty due to internal variability alone, which is irreducible, across timescales). An earlier study^43^ shows “mildly significant” increases in the observed US interannual precipitation variability. We calculate observed daily variability change over the second half of the 20^th^ century from station observations (Fig. 5). Precipitation variability has increased at most stations (though this increase in variability is similar in magnitude to the increase in mean precipitation), consistent with model projections of increased variability with warming. The median observed change is 13% K^−1^, compared to 8.0% K^−1^ during the same period in CMIP5 models, subsampled at grid boxes with observing stations.

## Discussion

In summary, precipitation variability simulated by climate models from daily and monthly to interannual and decadal timescales increases robustly over almost all land areas in response to anthropogenic warming in most models. For longer timescales, particularly interannual and longer, the forced increase may well be compensated for or amplified by natural variability (Fig. S5). We also show that observed daily precipitation variability increased over the second half of the 20^th^ century. In most models, regions, and seasons, the change in precipitation variability (measured by the mean standard deviation) is higher in magnitude than the change in mean precipitation and lower in magnitude than the change in moisture and extreme precipitation (heaviest day of rainfall each year). In most regions and seasons, mean precipitation increase sets a lower bound on the change in precipitation variability; the increase in precipitation variability is at least as large and in many cases greater than the increase in mean precipitation. We attribute this to the role of increasing moisture counteracted by weakening circulation.

In our analysis, we quantify precipitation variability by its standard deviation, and we examine its change in a set of model simulations subject to just one forcing scenario. While the standard deviation is a crude metric of precipitation variability, especially on short timescales with many dry periods, precipitation variability also increases when quantified by other metrics (Fig. S8). The standard deviation has the advantage of being well-defined, with a straightforward behavior in response to simple scaling of the distribution. Our analysis focuses on model simulations driven by a large increase in greenhouse gas forcing along with changes in aerosol forcing. Changes in variability will differ with the type of forcing, e.g., in an extreme example of geoengineering by stratospheric sulfate aerosol injection, where global-mean temperature does not change but mean precipitation decreases^44^. Finally, in our interpretation we have not considered mechanisms which might be important regionally, like soil moisture feedbacks.

Aggregated globally and over long timescales, the increased precipitation variability from daily all the way to decadal timescales is a robust emergent aspect of the water cycle that is changing as a result of anthropogenic warming, yet has received little attention. Both theoretical work and impact studies are required to complete and connect the understanding of changes in mean, variability, and extreme precipitation, as well as the risks they pose to society.

## Methods

### Model simulations

Model simulations include the CMIP5 multi-model ensemble^45^ (the r1i1p1 member only, 37 models with monthly data and 34 with daily data), CESM1 large ensemble^46^ (40 members), and GFDL ESM2M large ensemble^47^ (30 members). For all ensemble simulations, fully coupled simulations are forced by historical and RCP8.5 scenarios. We used output starting from 1951 because the GFDL large ensemble starts in 1950 and this allows for 1 year of spinup, and ending in 2100 when the GFDL and CESM1 simulations end.

### Standard deviation

We quantify precipitation variability by its standard deviation. We remove the linear trend from each time series before calculating its standard deviation. This largely removes the effect of transient warming at the end of the 21^st^ century in the RCP8.5 scenario.

### Extreme precipitation

We define extreme precipitation (shown in Figs 2 and 3) as the maximum daily precipitation each year (rx1day^48^), averaged over the same time periods as mean precipitation and precipitation variability.

### Statistical significance

Error bars or envelopes in Figs 1 and 4 show the 95% confidence interval of the multi-model mean calculated from a two-tailed Student’s *t*-test, using the standard deviation among ensemble members where the degrees of freedom is the number of ensemble members.

### Robustness across climate models

A change is considered “robust” when at least 67% of climate models agree on the sign of the change.

### Spatial aggregation

Spatial aggregation follows ref.^29^. We use each model’s own land mask and grid cell area fields to calculate the land fraction contributed by each grid cell. Where summer and winter seasons are identified, summer seasons are defined as June to August (JJA) north of the equator and December to February (DJF) south of it; vice versa for winter. Tropics and extratropics are defined as latitudes less and greater than 30°, respectively. Internal variability is quantified by comparing two members of the CESM1 ensemble drawn randomly without replacement during the base period.

### Regridding

For maps of the CMIP5 multi-model mean, calculations (mean, standard deviation, or extreme precipitation) are made on each model’s grid, data are regridded to a common 2.5 × 2.5° grid, the multi-model mean initial field is calculated, each model’s change is scaled by its change in global-mean surface air temperature, and then the multi-model mean absolute change is divided by the initial multi-model mean.

### Spatial averaging

For Figs 3 and 4, the spatial-average change in each of mean precipitation, standard deviation of precipitation, or mean moisture is calculated by first calculating the variable at each grid cell. In the case of standard deviation, it is squared. Then, the spatial average of the variable for the initial and final time periods is calculated, and in the case of standard deviation, the square root is taken. Next, the difference of the spatial average between the time periods is calculated, followed by scaling by each model’s global-mean surface air temperature change over the appropriate period of time (in Fig. 4), and finally calculating the multi-model mean.

### Timescale dependence

For each timescale, the standard deviation is calculated for an initial and final time period. Spatial averaging is carried out as described above. For daily through annual timescales, the initial period is 1976–2005 and the final period is 2071–2100. Daily data for 34 CMIP5 models are used. For the 1-month timescale, the annual cycle is removed before the standard deviation is calculated. For the 3-month timescale, monthly data with annual cycle removed are averaged in 3-month seasons and the standard deviation is calculated. For the 1-year timescale, monthly data is averaged over years. For the 3-year timescale, monthly data are averaged in 3-year periods over the 51-year periods 1955–2005 and 2050–2100 to achieve sufficient sampling.

### Observational analysis

Daily precipitation station data come from the Global Historical Climatology Network Daily^49^ dataset, version 3.22^50^ and use the subset of stations in the Global Climate Observing System (GCOS) Surface Network (GSN). This subset of stations has better spatial sampling than the total network. Only stations with data for at least 50% of each period are used. Global-mean surface temperature observations are calculated from NOAA GlobalTemp^51,52^. Change in the standard deviation of daily precipitation is calculated from 1960–1969 to 1990–1999. The time periods are chosen to maximize the number of stations; we repeated the analysis for start dates in the 1950s and 1970s and end dates from the 1990s through 2016, with similar results.

### Data availability

CMIP5 simulations are available from PCMDI through the Earth System Grid (ESG) repository at https://esgf-node.llnl.gov/projects/cmip5/. CESM1 simulations are available from the ESG at https://www.earthsystemgrid.org/dataset/ucar.cgd.ccsm4.CESM_CAM5_BGC_LE.html. GFDL large ensemble data can be requested from the corresponding author upon reasonable request and with permission of K.R. Rodgers. GHCN-Daily data are available from NOAA at 10.7289/V5D21VHZ.

## Electronic supplementary material

Supplementary Information
