# Supplementary material for: Precipitation variability increases in a warmer climate
Source: Sci Rep. 2017 Dec 21;7:17966. doi: 10.1038/s41598-017-17966-y (PMC5740108; doi:10.1038/s41598-017-17966-y)
Supplement: Supplementary file 1 — Supplementary Information [file 41598_2017_17966_MOESM1_ESM.pdf]

Supplementary Information for “Precipitation variability increases in a warmer climate”

Angeline G. Pendergrass*[1], Reto Knutti[1,2], Flavio Lehner[1], Clara Deser[1], Benjamin M. Sanderson[1]

[1]National Center for Atmospheric Research, Boulder, CO, USA

[2] Institute for Atmospheric and Climate Science, ETH Zurich, CH-8092 Zurich, Switzerland

*Corresponding author address: Angeline G. Pendergrass, P.O. Box 3000, Boulder, CO 80307. Email: apgrass@ucar.edu

Supplementary Text

Power spectral density change

The seasonal power spectral density is calculated for monthly data from 1963-2005 and 2058-2100, each smoothed with a Hamming window. The daily power spectral density is calculated from 1976-2005 and 2071-2100 using 256 day windows smoothed with a Hamming window with an overlap of 128 days. Each difference is scaled by the change in global-mean surface air temperature over the relevant time periods.

ENSO analysis

Following Ref. 1, ENSO is defined as the first principal component of deseasonalized monthly surface air temperature between 30 N to 30 S during 50 years periods, 1951-2000 and 2051-2100. Three-month (seasonal) means of this principal component are calculated, and those whose magnitude is less than 0.5σ are considered neutral. The 0.5σ threshold is calculated separately for each model in the CMIP5 archive, each single-model ensemble, and separately for each 50-year period.

Timescale dependence

Following Fig. 4, described in methods. Additionally, two members of the CESM single-model ensemble have hourly precipitation output; 5-year periods 1988-1992 and 2083-87 are used. Daily data for 40 CESM ensemble members are used. For the 30 y timescale, the initial period is 1976-2005 and the final period is 2071-2100. For the 10, 30, and 50-y timescales, the standard deviation across each single-model ensemble is calculated. For

the 10-y timescales, three decades of the period are included. For 50 y the periods used are 1956-2005 and 2051-2100.

Alternative metrics for variability

We examined alternative metrics for precipitation variability for the daily and seasonal timescales. First, we tested the effect of three adjustments to the timeseries before calculation of standard deviation: isolating only wet days (instead of all days), not removing the seasonal cycle (instead of removing it), and not detrending timeseries (instead of detrending it). The details of the data treatment before the standard deviation is calculated have very little effect on the magnitude of the change in standard deviation.

Second, we calculated the difference between 50th and 84.1, 97.7, and 99.8th percentiles of precipitation, which would be equivalent to 1, 2, and 3 standard deviations of change for a gaussian variable. The magnitude of change of standard deviation falls between the changes of the width measured from 50th to 97.7 and 99.8th percentiles. For most metrics, the difference in magnitude of change is larger for daily than for seasonal timescales. By all measures except daily deseasonalized 84.1-50th percentile, the precipitation variability increases with warming with 95% confidence according to a two-tailed student's *t*-test across the multi-model ensemble.

Finally, we fitted the width parameter for the maximum likelihood fit to the wet-day lognormal distribution. The parameters of the lognormal distribution are fit at each grid point and then averaged in space. For daily data, there is a small but significant increase

in the width of the lognormal distribution averaged over land; the change in lognormal width in the global mean is not distinguishable from zero (not shown). For seasonal data, the increase in lognormal width averaged over land increases. In all cases, the change is smaller than the changes in the standard deviation. The standard deviation of precipitation (and other measures of the width of the distribution in linear space) is related to the lognormal distribution by not only by its width, but also its location parameter. The linear width, rather than the lognormal one, is probably the more physically-relevant quantity for many impacts. Of course this list is not exhaustive; still other metrics are possible. For example, the Gini coefficient has been used to measure the temporal uniformity of precipitation[2], which is non-uniquely related to the variability.

Supplementary Figures

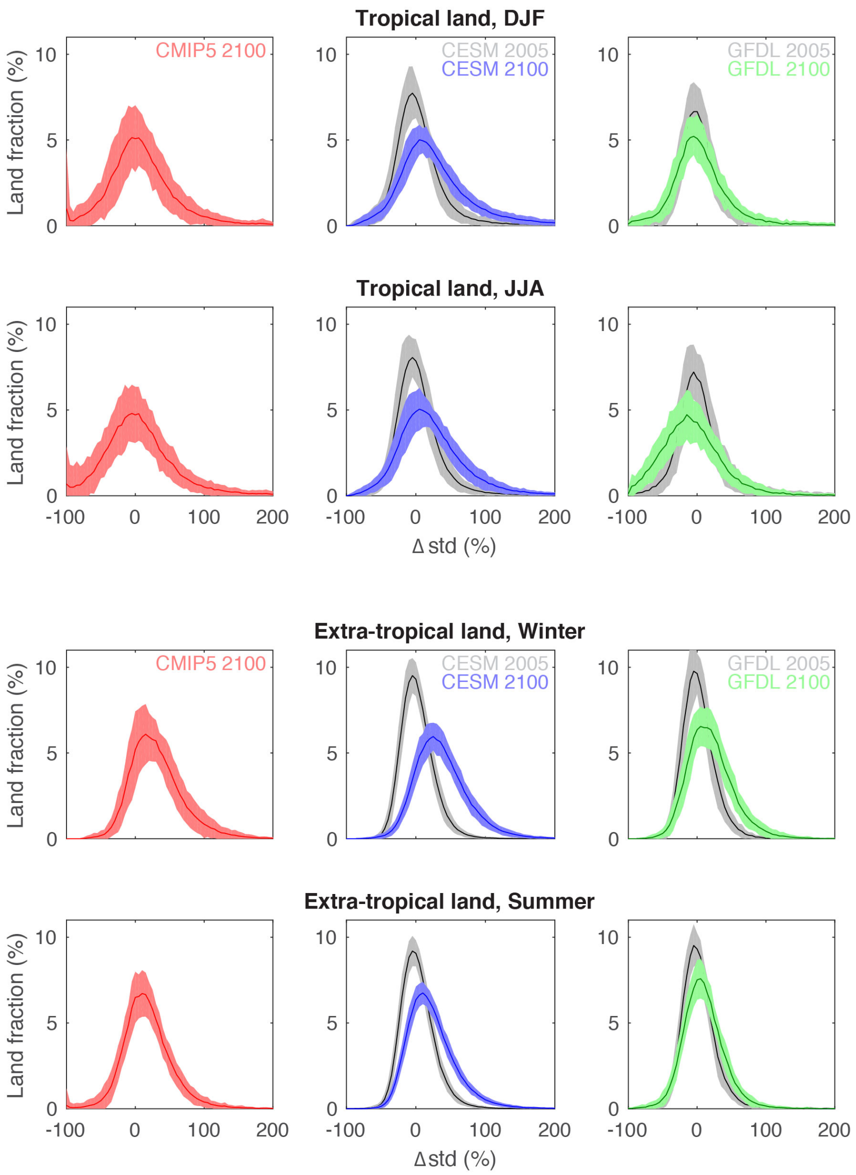

Figure S1. Spatially aggregated precipitation variability change. Land area fraction experiencing a given change in the standard deviation of seasonal mean precipitation at individual grid points 2071-2100 relative to 1976-2005, and comparison between randomly-drawn members of each single-model ensemble for 1976-2005 as an estimate of changes expected from natural variability.

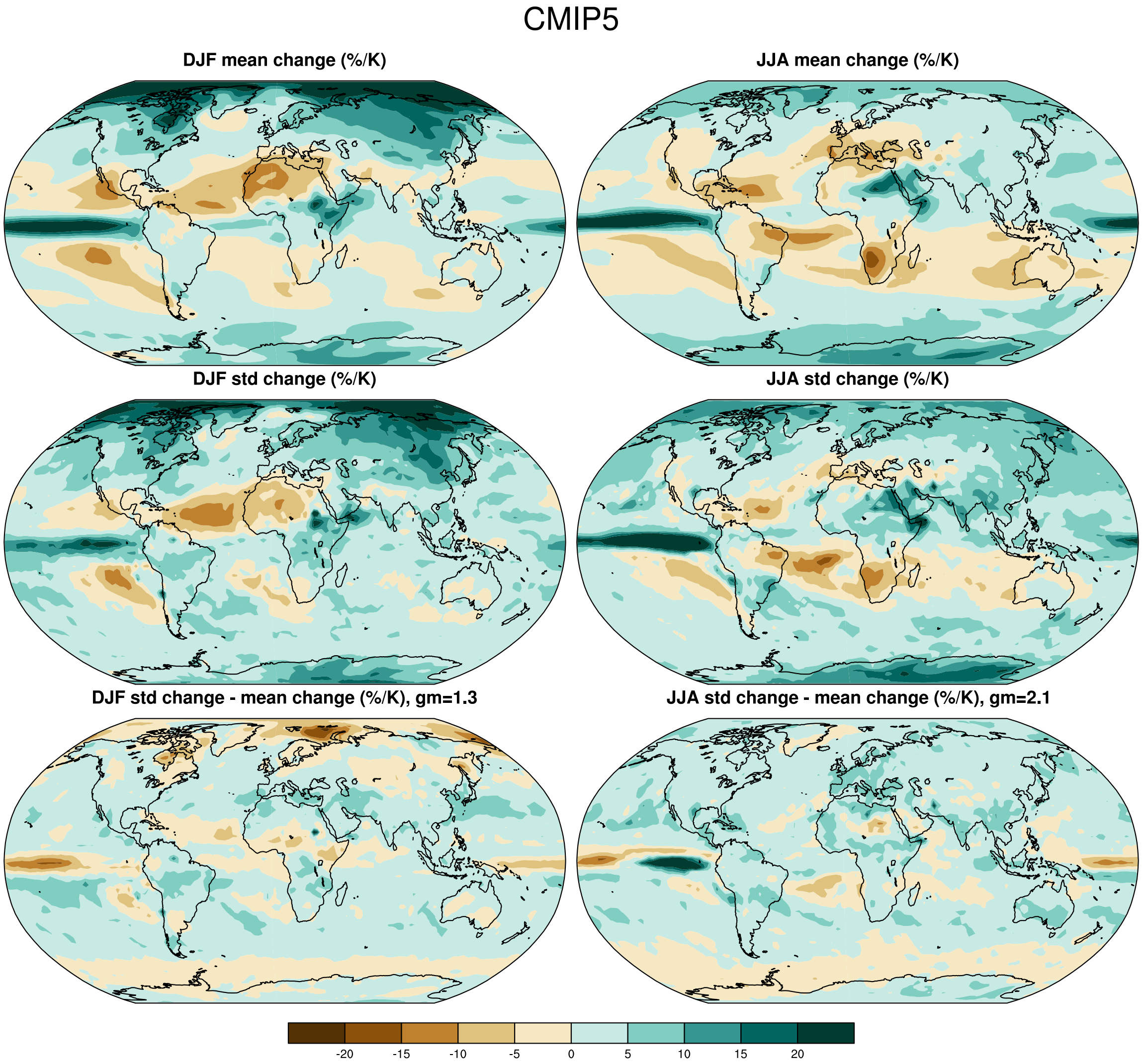

Figure S2. Spatial patterns of seasonal changes in mean and standard deviation. Change in precipitation mean and standard deviation of seasonally-averaged precipitation for the CMIP5 multi-model mean in 2071-2100 relative to 1976-2005, scaled by the change in global mean surface air temperature, and the difference between variability and mean precipitation change. Maps were generated using NCL[3].

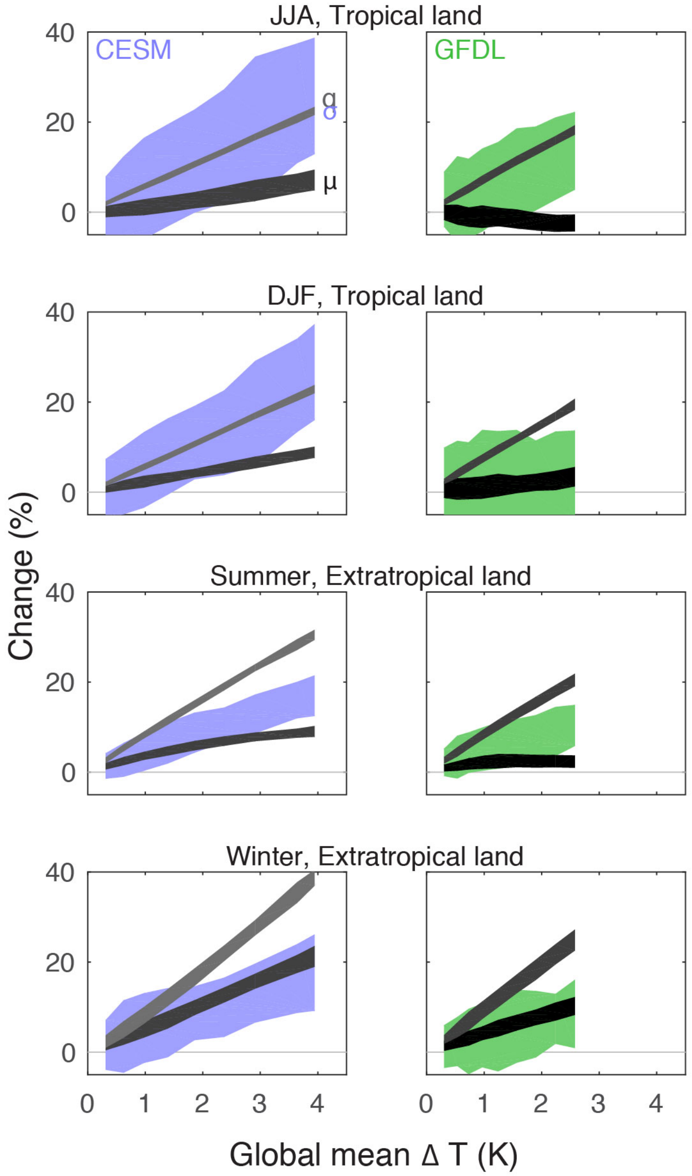

Figure S3. Change in mean precipitation, precipitation variability, and moisture. Change in mean precipitation, interannual precipitation variability, and moisture following Fig. 3 for the CESM and GFDL initial-condition ensembles. Envelopes indicate 95% confidence intervals across each ensemble.

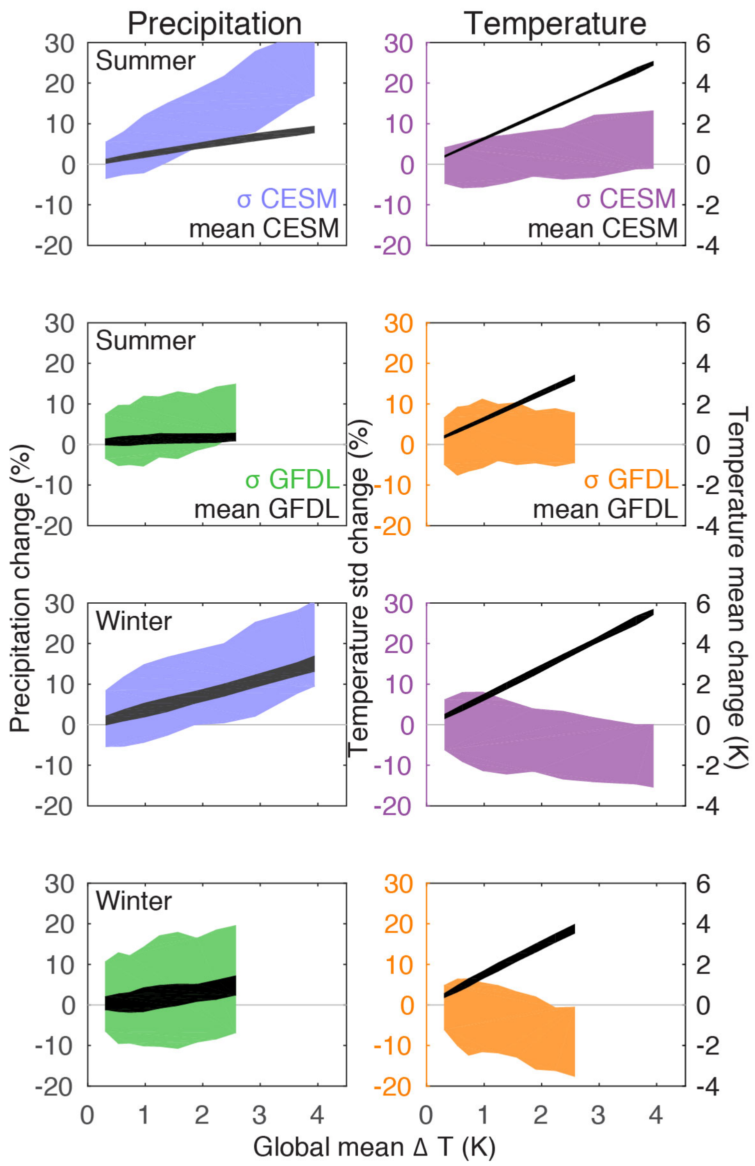

Figure S4. Change in mean and variability of precipitation and temperature. Change in mean and interannual standard deviation of precipitation and temperature for CESM and GFDL single-model ensembles over global land. Envelopes indicate 95% confidence intervals across each ensemble.

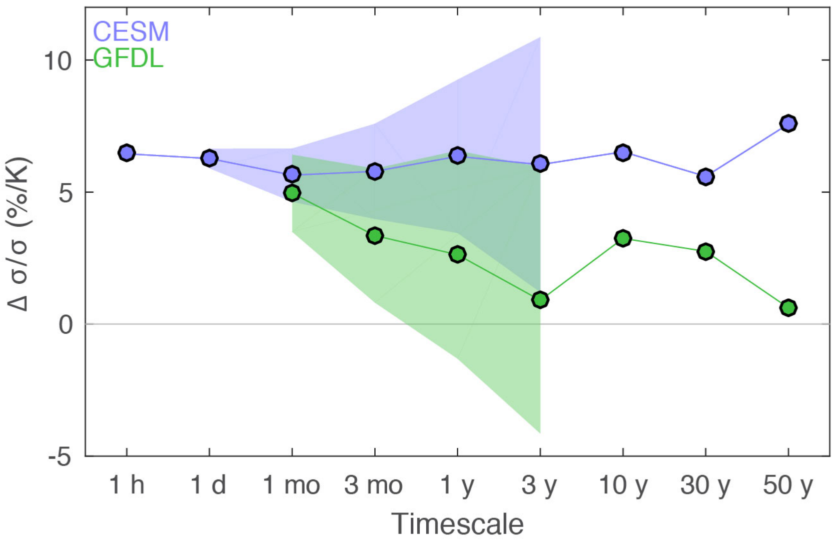

Figure S5. Precipitation variability change by timescale. Change in standard deviation of precipitation averaged over land per degree global-mean surface air temperature change over the 21st century (see Supplementary Text for exact time periods) for the CESM and GFDL ensembles. Error bars indicate 95% confidence.

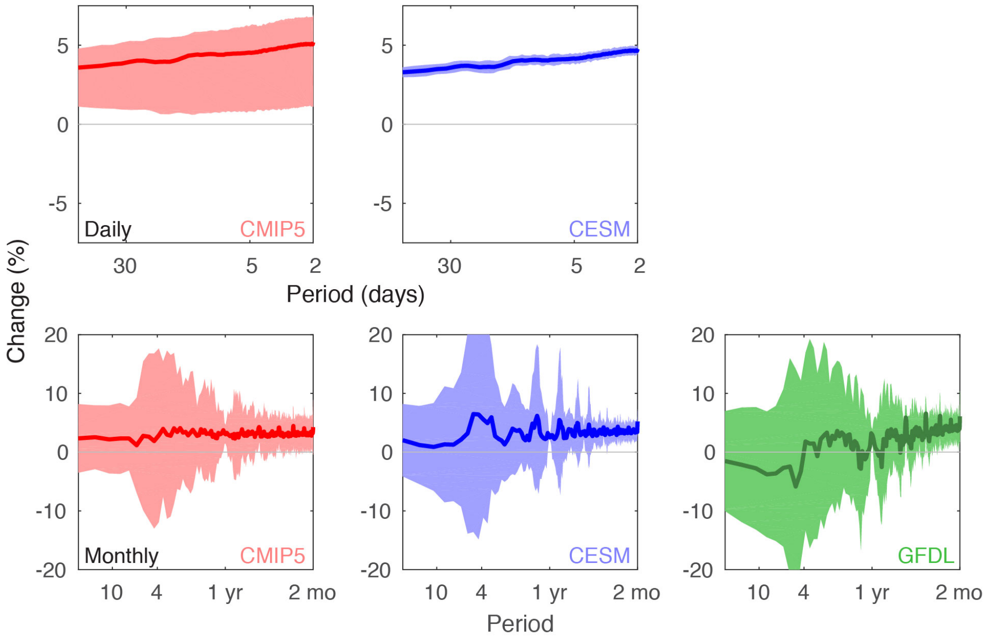

Figure S6. Change in power spectral density. Ensemble and globally-averaged change in power spectra of daily and monthly data with warming for CMIP5, CESM, and GFDL ensembles. Error bars indicate 95% confidence. See Supplementary Text for details.

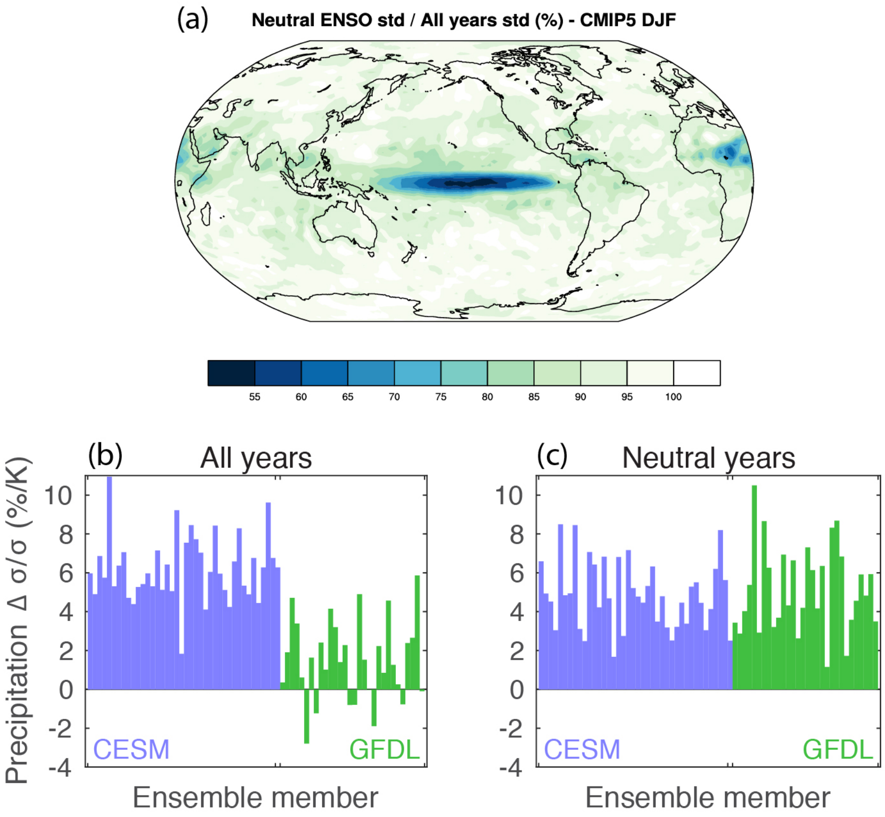

Figure S7. Influence of ENSO on interannual precipitation variability. (a) Ratio of the standard deviation of DJF-mean precipitation from ENSO-neutral years to all years in the CMIP5 multi-model mean (see Supplementary Methods for details). The map was generated using NCL[3]. Change in tropical land-averaged standard deviation change for each single-model ensemble member for (b) all years and (c) ENSO-neutral years.

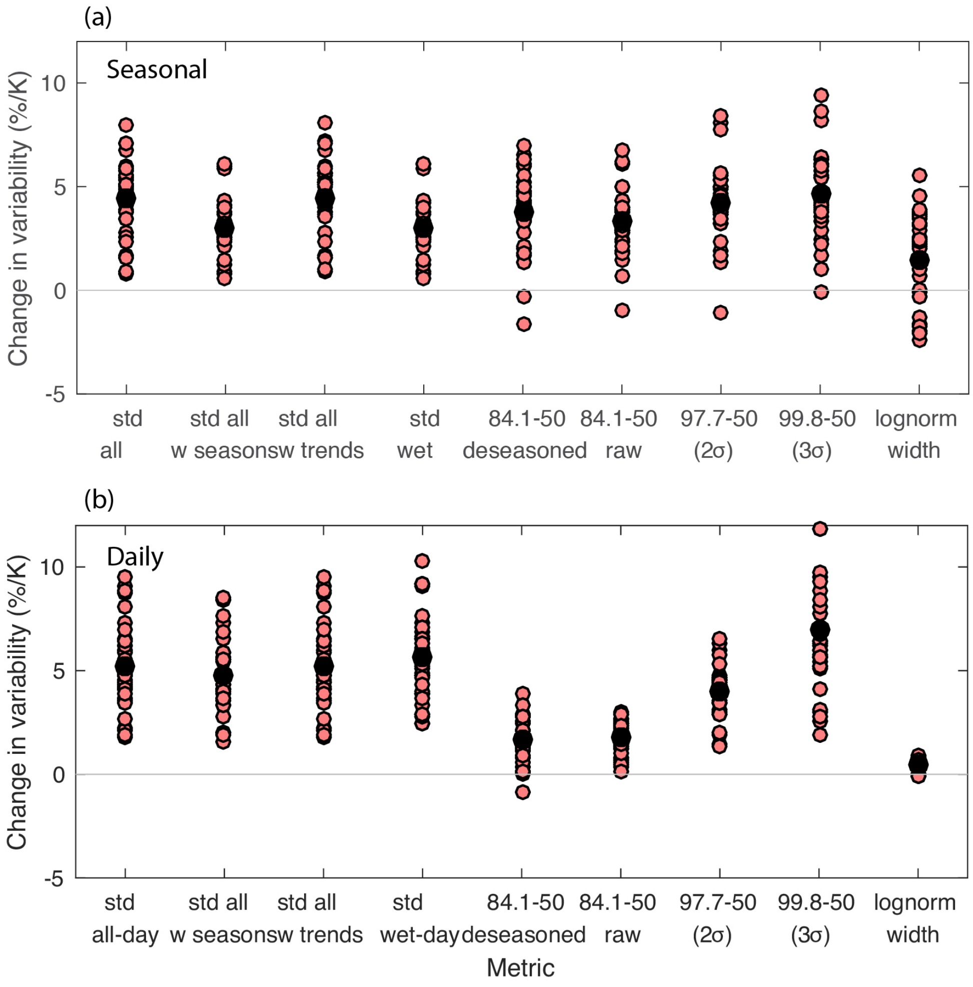

Figure S8. Alternative metrics for precipitation. Standard deviation calculated from all times, without deseasonalizing, without detrending, and only times with precipitation; the distance between 84.1 and 50th percentiles (with and without deseasonalizing), 97.7-50 and 99.8-50 (without deseasonalizing), and the width of the fitted lognormal distribution. Each CMIP5 model's change averaged over all land grid points is shown in red, and the multi-model mean is shown in black. The difference is calculated from 1976-2005 to

2071-2100 in the RCP8.5 forcing scenario. Alternative metrics are calculated for (a) seasonal and (b) daily precipitation timeseries.

References

1. Power, S., Delage, F., Chung, C., Kociuba, G. & Keay, K. Robust twenty-first-century projections of El Niño and related precipitation variability. *Nature* **502,** 541–5 (2013).
2. Konapala, G., Mishra, A. & Leung, L. R. Changes in temporal variability of precipitation over land due to anthropogenic forcings. *Environ. Res. Lett.* **12,** 24009 (2017).
3. The NCAR Command Language (NCL), Version 6.3.0. (2015). doi:10.5065/D6WD3XH5
